# AG-205 Upregulates Enzymes Involved in Cholesterol Biosynthesis and Steroidogenesis in Human Endometrial Cells Independently of PGRMC1 and Related MAPR Proteins

**DOI:** 10.3390/biom11101472

**Published:** 2021-10-06

**Authors:** Charlotte Thieffry, Marie Van Wynendaele, Asena Aynaci, Mauriane Maja, Caroline Dupuis, Axelle Loriot, Etienne Marbaix, Patrick Henriet

**Affiliations:** 1 CELL Unit, de Duve Institute and Université Catholique de Louvain, B-1200 Brussels, Belgium; charlotte.thieffry@uclouvain.be (C.T.); marie.vanwynendaele@uclouvain.be (M.V.W.); asena.aynaci@unamur.be (A.A.); mauriane.maja@uclouvain.be (M.M.); caroline.dupuis@student.uclouvain.be (C.D.); etienne.marbaix@uclouvain.be (E.M.); 2 GEPI Unit, de Duve Institute and Université Catholique de Louvain, B-1200 Brussels, Belgium; axelle.loriot@uclouvain.be; 3 Pathology Department, Cliniques Universitaires Saint-Luc, B-1200 Brussels, Belgium

**Keywords:** PGRMC1, endometrium, AG-205, cholesterol, steroidogenesis

## Abstract

An inappropriate response to progestogens in the human endometrium can result in fertility issues and jeopardize progestin-based treatments against pathologies such as endometriosis. PGRMC1 can mediate progesterone response in the breast and ovaries but its endometrial functions remain unknown. AG-205 is an alleged PGRMC1 inhibitor but its specificity was recently questioned. We added AG-205 in the cultures of two endometrial cell lines and performed a transcriptomic comparison. AG-205 significantly increased expression of genes coding enzymes of the cholesterol biosynthetic pathway or of steroidogenesis. However, these observations were not reproduced with cells transfected with siRNA against PGRMC1 or its related proteins (MAPRs). Furthermore, AG-205 retained its ability to increase expression of selected target genes even when expression of PGRMC1 or all MAPRs was concomitantly downregulated, indicating that neither PGRMC1 nor any MAPR is required to mediate AG-205 effect. In conclusion, although AG-205 has attractive effects encouraging its use to develop therapeutic strategies, for instance against breast cancer, our study delivers two important warning messages. First, AG-205 is not specific for PGRMC1 or other MAPRs and its mechanisms of action remain unclear. Second, due to its effects on genes involved in steroidogenesis, its use may increase the risk for endometrial pathologies resulting from imbalanced hormones concentrations.

## 1. Introduction

An appropriate response to progesterone is crucial for the physiology of female reproductive organs. In addition to the well-documented nuclear progesterone receptors, two other families of proteins have been the subject of growing interest for their potential to mediate progesterone response: the class II progestin and adipoQ receptors (PAQRs, also called mPRs) and the membrane-associated progesterone receptors (MAPRs). The latter family contains four members sharing a cytochrome b5-like heme-binding domain on which their bioactivity depends [1]: progesterone receptor membrane component-1 and -2 (PGRMC1 and PGRMC2), neudesin (encoded by the *NENF* gene, for *neudesin neurotrophic factor*) and neuferricin (encoded by the *CYB5D2* gene for *cytochrome b5 domain containing 2*): among MAPRs, PGRMC1 received the greatest attention because it is suspected to participate in, or control, a large range of biological functions. including cell proliferation, apoptosis, steroid metabolism, lipid metabolism, membrane trafficking, angiogenesis, and progesterone response. Moreover, PGRMC1 overexpression is considered to play roles in carcinogenesis [2]. PGRMC1 is also required for optimal fertility. Indeed, in zebrafish, a double knockout of *PGRMC1* and *PGRMC2* resulted in decreased fertility due to a reduction in ovulation and a downregulation of the nuclear progesterone receptor protein [3]. Moreover, a conditional *Pgrmc1* knockout in a mouse uterus led to the appearance of multiple endometrial cysts and a decrease in the number of offspring [4]. PGRMC1 was also shown to favour human trophoblastic cell implantation [5].

A significant proportion of the studies investigating PGRMC1 have relied on the use of AG-205 (PubChem entry 1202545, CAS 1375078-57-1, UNII-02137X034H, IUPAC: 1-((4aR,9bS)-2,8-Dimethyl-3,4,4a,9b-tetrahydro-1H-pyrido(4,3-b)indol-5-yl)-2-(1-(4-chlorophenyl)tetrazol-5-yl)sulfanyl-ethanone). AG-205 is a small molecule (Figure S1) commercialized by major biotech companies as a PGRMC1 inhibitor/ligand, although evidence is lacking to support this assumption. AG-205 was initially identified as one of four aromatic molecules able to bind the *Arabidopsis thaliana* AtMAPR2 [6] aka AtMP3 (UniProt entry Q9SK39). Similar to the four MAPR proteins, AtMAPR2/AtMP3 contains a cytochrome b5-like heme-binding domain and, more precisely, two key tyrosine residues (positions 107 and 113 in PGRMC1) required for heme binding [7]. Because the addition of AG-205 to purified PGRMC1 modified the spectroscopic properties of the PGRMC1-heme complex and induced dissociation of heme-dependent PGRMC1 homodimers, it was assumed that PGRMC1 was the human orthologue of yeast AtMAPR2/AtMP3 [8]. It is not clear whether the ability of AG-205 to alter the spectrometric properties of the other MAPRs was tested. Moreover, comparison of the AtMAPR2/AtMP3 protein sequence with that of the 4 human MAPRs (entry O00264 for PGRMC1, O15173 for PGRMC2, Q9UMX5 for neudesin and Q8WUJ1 for neuferricin), using the Clustal Omega Multiple Sequence Alignment tool from EMBL-EBI does not support a closer homology between AtMAPR2/AtMP3 and PGRMC1 than with the three other MAPRs.

In the ovary and the breast, the addition of AG-205 promoted apoptosis [9,10] modified regulation of the cell cycle [10,11,12] and reduced cell migration and invasion capacities [10]. As a consequence, AG-205 was patented for its therapeutic potential against breast cancer [13]. However, to the best of our knowledge, AG-205 is only used for research purposes, and although PGRMC1 was proposed to be an important regulator of essential pathways in the breast and ovary, much less is known about its endometrial functions and mechanisms of action. In the human endometrium, progesterone is a critical inducer of modifications occurring to favour blastocyst implantation and pregnancy, including decidualization, i.e., a specific differentiation of the endometrial stromal cells. PGRMC1 is expressed in the human endometrium and its potential contribution to decidualization was recently reported [14]. Interestingly, the addition of AG-205 to endometrial stromal cells undergoing artificial decidualization in response to progesterone (combined with estradiol) upregulated expression of genes related to metabolism, molecular transport and hormonal biosynthesis. However, it is unclear whether these changes required progesterone. Indeed, direct PGRMC1 binding to progesterone remains highly debated. Alternatively, the presence of potential SH2- and SH3-binding domains strongly suggests that PGRMC1 can act as a “hub” protein, connecting multiple partners [15] to activate molecular pathways that are—directly or indirectly—sensitive to progesterone. Moreover, and more importantly, two recent publications have strongly challenged AG-205 specificity towards PGRMC1 (and PGRMC2). Firstly, knocking out *PGRMC1* and/or *PGRMC2* expression did not alter the ability of AG-205 to induce the formation of large endosomes in CHO-K1 and HeLa cells [16]. Secondly, and through a more direct approach, no binding activity of AG-205 to apo- or heme-dimerized PGRMC1 was observed by isothermal titration calorimetry analysis [17].

In the present study, we first used a transcriptomic approach to identify biological processes and individual genes impacted by the addition of AG-205 in two endometrial cells lines cultured in the absence of progesterone. We then compared these transcriptomes with those derived from the same endometrial cells transfected with siRNAs directed against *PGRMC1* or against the four MAPRs. In both cell lines, the addition of AG205 increased expression of genes involved in sterol biosynthesis and steroidogenesis, as previously reported, but this effect was independent of the presence of progesterone and of the 4 MAPRs.

## 2. Materials and Methods

### 2.1. Cell lines and Cell Culture

Two human endometrial cell lines were used for the experiments: the Telomerase-immortalised Human Endometrial Stromal Cell line (T-HESC, ATCC CRL-4003) derived from fibroblast-like cells obtained from an adult patient with myomas [18], and the Human Endometrial Cancer One A cell line (HEC-1A, ATCC HTB-112) derived from epithelial-like cells isolated from a patient with stage 1A endometrial adenocarcinoma [19].

Cells were grown in Dulbecco’s Modified Eagle Medium/Nutrient Mixture F-12 (DMEM/F12; Gibco, ThermoFisher Scientific, Merelbeke, Belgium), supplemented with 10% Fetal Bovine Serum (FBS), 100 U/mL penicillin, 100 µg/mL streptomycin (ThermoFisher Scientific) in a humidified atmosphere of 5% CO_2_ at 37 °C.

### 2.2. Chemical Compounds

AG-205 (Sigma, Saint-Louis, MO, USA) was diluted in dimethyl sulfoxide (DMSO) to prepare a 15 mM (1000×) stock solution.

### 2.3. Cell Viability Assay

The optimization of the final concentration and the incubation time of AG-205 was carried out with the CellTiter 96^®^ AQueous One Solution Cell Proliferation Assay (Promega, Leiden, The Netherlands) according to the manufacturer’s recommendations. Briefly, cells were seeded in 96-well plates (2 × 10^4^ cells/mL) and grown in DMEM/F12, without phenol red nor antibiotics, supplemented with 10% FBS. After 48 h incubation, medium was changed after supplementation with indicated concentrations of AG-205 or corresponding DMSO concentration as control. Cells were incubated for 24 h, 32 h or 48 h before the addition of 20 µL/well of CellTiter 96^®^ AQueous One Solution Reagent containing a tetrazolium compound (MTS). After 1–4 h incubation at 37 °C, the quantity of formazan (a bio-reduced colored product of MTS directly proportional to the number of living cells) was measured at 490 nm absorbance.

### 2.4. Inhibition Techniques (siRNA Transfection or AG-205 Addition)

siRNA-mediated gene silencing was performed by transient transfection with Lipofectamine RNAiMax (Invitrogen, Waltham, MA, USA) according to the manufacturer’s recommendations.

Cells (2 × 10^4^ cells/mL) were transfected with final 10 nM pre-designed Silencer siRNA(s) or negative control (Table S1, Supplementary Materials) and cultured in DMEM/F12, without phenol red nor antibiotics, supplemented with 10% FBS. At the same time, additional wells were prepared and cultured with the same medium (without transfection) for the AG-205 inhibition strategy. After 48 h incubation, the medium of all wells was changed and 15 µM of AG-205 or 0.1% DMSO, as negative control, was added to the appropriate wells for an additional 32 h.

### 2.5. RNA Extraction

After incubation, cells were lysed with TRIzol Reagent (Ambion, Huntingdon-Cambridgeshire, UK), vortexed for 10 s and incubated for 10 min at room temperature. The lysates were vortexed during 30 s and 20 ng of tRNA were added to each sample to stabilize the RNA. After homogenization, 200 µL of chloroform was added. The mixtures were vortexed for 30 s and incubated for 15 min at room temperature. After centrifugation for 15 min at 12,000× *g* and 4 °C, upper aqueous layers were collected. Samples were supplemented with 200 µL isopropanol 100%, vortexed for 30 s and stored for at least 1 h at −80 °C to precipitate the RNAs. After a 30 s centrifugation at 12,000× *g* and 4 °C, the RNA pellet was washed with cold ethanol 70%, dried at room temperature and resuspended in autoclaved H_2_O_d_. For RNA sequencing, an additional step was performed to remove excess DNA with the TURBO DNA-free kit (Invitrogen) according to manufacturer’s recommendations except that a longer centrifugation step (3 min) was carried out.

### 2.6. Quantitative Real-Time PCR

For all samples, the RNA concentrations were evaluated using Nanodrop ND-8000 spectrophotometer. Then, 500 ng total RNA were used for reverse transcription using SuperScript III Reverse Transcriptase kit (Invitrogen), according to the manufacturer’s recommendations.

Primers for amplification of *PGRMC1* were previously published [20]. All other specific oligonucleotides were designed in our laboratory (Table S2) and their amplification efficiencies were checked before use. The real-time PCR amplifications were performed with the KAPA SYBR FAST qPCR Master Mix (2×), 0.25 µM primers (forward and reverse) and 15 ng cDNA. Two HouseKeeping Gene, RPL13a and β-actin were selected based on their stability of expression under our experimental conditions.

### 2.7. RNA Sequencing

Total RNA was isolated from three independent cell culture experiments. The RNA quantities and qualities were evaluated using a Nanodrop ND-8000 spectrophotometer and an Agilent Bioanalyzer, respectively. RNA library preparation was performed using a polyA selection method. RNA sequencing was performed using the Illumina HiSeq system in a 2 × 150-bp configuration (single index, per lane) by GENEWIZ. RNA sequencing data of cells cultured with AG-205 are stored under GEO accession number GSE174305. RNA sequencing of cells cultured with the siRNA-PGRMC1 (s21310) were only used for the purpose of comparison and will be commented on in detail in another publication.

### 2.8. Bioinformatics Analysis Workflow

Read quality control was performed using FastQC software v0.11.8 [21]. Low quality reads were trimmed, and adapters were removed using Trimmomatic software v0.38 [22]. Reads were aligned using HISAT2 software v2.1.0 [23] on GRCh38 genome. Gene counts were generated using featureCounts software from subread-2.0.0 [24] and Ensembl Homo_sapiens.GRCh38.94.gtf annotation file. Differential expression analyses were done using DESeq2 v1.30 [25], on R version 4.0.3. Genes with adjusted *p*-value < 0.05 and absolute log2 fold-change > 1 were considered as differentially expressed. Over Representation Analysis was done with clusterProfiler v3.16.1 bioconductor package.

### 2.9. Immunolabeling

Immunocytofluorescence was performed with cells cultured on glass coverslips. After appropriate incubation, cells were washed in PBS before fixation for 10 min in 4% paraformaldehyde. They were washed 5 times for 5 min in PBS and permeabilized for 15 min in PBS with 0.1% Triton X-100. Nonspecific binding sites were blocked for 1 h at room temperature with PBS, 0.3% Triton X-100, 5% normal goat serum before incubating coverslips overnight at 4 °C with 1/200 anti-PGRMC1 primary antibody (D6M5M, cat no. 13856; Bioké, Leiden, The Nederlands). The next day, cells were washed 3 times for 5 min in PBS and incubated with 1/1000 secondary antibody (Goat anti-rabbit IgG, Alexa 488; Life Technologies, Merelbeke, Belgium) for 1 h 30 at room temperature. Nuclei were counterstained with Hoechst (BisBenzimide H33342, 1 µg/mL; Sigma) during the incubation with the secondary antibody. Fluorescence was observed with a Cell Observer Spinning Disk (COSD) confocal microscope (Zeiss, Zaventem, Belgium). Signals were analysed and quantified with the image analysis platform HALO (Indica Labs, Albuquerque, NM, USA).

### 2.10. Cell Fractionation and Western Blot Analysis

Cells incubated with AG-205 or DMSO control were washed with PBS, lysed with cytoplasmic lysis buffer (50 mM Tris, 0.1% Nonidet P-40 (Igepal CA-630), supplemented with Complete protease inhibitor cocktail (1 tablet for 50 mL; Roche/Boehringer, Brussels, Belgium)) and incubated for 30 min on ice. The samples were centrifuged for 10 min at 14,000× *g* and 4 °C to separate cytoplasmic (supernatant) and nuclear (pellet) proteins. The pellets were washed 3 times before the addition of nuclear lysis buffer (0.1% SDS, 1% sodium deoxycholate, 0.5% NP-40 (Tergitol), supplemented with Complete protease inhibitor cocktail (1 tablet for 50 mL)) and incubation for 30 min under intense shaking. To complete lysis, the homogenates were successively passed through a 16 G and a 30 G syringe and sonicated. The nuclear fraction was isolated (supernatant) after a last centrifugation step.

Cells transfected with siRNA-PGRMC1, or siRNA-control were washed with PBS and lysed with RIPA buffer (50 mM HEPES, 50 mM NaCl, 0.5% sodium deoxycholate, 0.1% SDS, 0.5% octylglucoside, supplemented with Complete protease inhibitor cocktail (1 tablet for 50 mL)).

All lysates were sonicated, and the protein concentration was measured by the bicinchoninic acid (BCA) method. When necessary, samples were concentrated with Amicon Ultra-0.5 Centrifugal Filter Units (MerckMillipore, Overijse, Belgium), according to the manufacturer’s recommendations. Next, sample buffer 5× (0.25 M Tris-HCl, 10% SDS, 20% Glycerol, 0.005% Bromophenol Blue, 5 mM DTT, pH 6.8) was added to each sample and the homogenates were heated for 5 min at 100 °C and centrifuged for 5 min at 14,000 g. Samples were prepared to ensure corresponding amounts of biomaterial in compared conditions (DMSO versus AG-205; siCTRL vs. siPGRMC1). Proteins were separated by SDS-PAGE (Running Buffer: Tris 0.025 M, glycine 0.192 M, SDS 0.1%) on a 12% polyacrylamide gel and transferred to a PVDF membrane (Perkin-Elmer, Zaventem, Belgium). Membranes were blocked for 2 h at room temperature with Tris Buffer Saline (TBS: 20 mM Tris-HCL, 0.5 M NaCl, pH 7.5), supplemented with 0.05% Tween-20 (TBST) and 5% non-fat milk, to avoid non-specific binding, before incubation overnight at 4 °C with the primary anti-PGRMC1 antibodies (D6M5M) diluted at 1:1000 in TBST, 5% bovine serum albumin (BSA).

After 3 washes of 10 min in TBST, blots were incubated for 1 h at room temperature with horseradish peroxidase-conjugated secondary antibodies (cat no. 050M4834; Sigma; or cat no. G21040; Life Technologies) diluted in TBST, 5% BSA. They were washed 3 times in TBST and once in TBS. Immunoreactive bands were revealed by chemiluminesence (SuperSignalTM West Femto Maximum Sensitivity Substrate; ThermoFisher Scientific) using the Fusion Solo S (Vilber Lourmat, Collegien, France).

For controls of equal loading, membranes were placed in a stripping buffer for 15′ (ReBlot Plus Strong Antibody Stripping Solution (10×); MerckMillipore), and re-probed with a primary anti-GAPDH (glyceraldehyde-3-phosphate dehydrogenase) antibody (catalog no. AM4300; Ambion, Huntingdon-Cambridgeshire, United Kingdom, as cytoplasmic control) diluted at 1:8000 in TBST, 5% non-fat milk and/or with a primary anti-histone H3 antibody (catalog no. Ab1791; Abcam, Amsterdam, The Nederlands, as a nuclear control) diluted at 1:10,000 in TBST, 5% BSA.

### 2.11. Statistical Analysis

All statistical analyses were performed with GraphPad Prism 8.4.3. Analyses of RT-qPCR data were performed on paired ΔCt values (i.e., within the same experiment). The outliers were identified and excluded using the Grubbs’ test.

## 3. Results

### 3.1. Setup of AG-205 Use in Endometrial Cell Culture

Our experiments were performed in two human endometrial cell lines, T-HESC from fibroblastic origin [18] and HEC-1A from epithelial origin [19]. Since AG-205 was used at quite high concentrations in previous publications (in the micromolar range), we first determined the effect of AG-205 addition on the viability of the two endometrial cell lines. HEC-1A and T-HESC cells were cultured for 24, 32 or 48 h in the presence of AG-205 (or DMSO as control) and cell viability was measured by colorimetric assay at the end of the culture (Figure S2, Supplementary Materials). In both cell lines, AG-205 had no effect on cell viability for up to 48 h when added at 5 or 15 µM. In contrast, cell viability was significantly reduced after 32 and 48 h upon addition of 30 µM AG-205, and at all time points upon addition of 45 µM AG-205. We therefore decided to perform all subsequent experiments with 15 µM AG-205 for 32 h.

**Figure 1:**
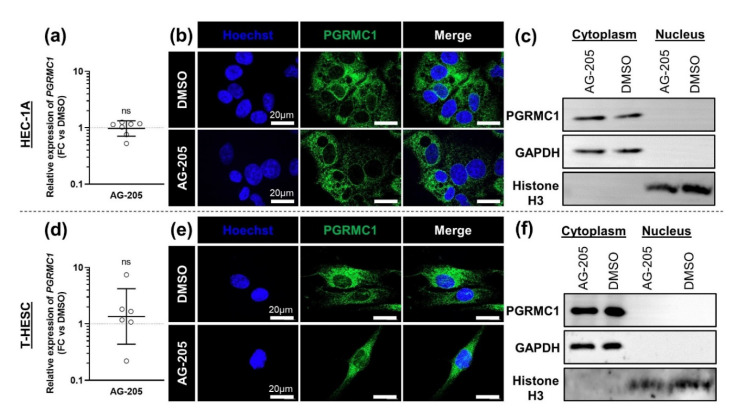
AG-205 does not modify expression of PGRMC1. Relative expression, immunolocalization and immunoblotting of PGRMC1 in HEC-1A (**a**–**c**) and T-HESC (**d**–**f**) cell lines treated with 15 µM AG-205 or control DMSO during 32 h. (**a**,**d**) Relative expression of *PGRMC1* was measured by RT-qPCR, normalized, compared to corresponding DMSO controls and is presented as individual fold changes (FC) in log scale and as geometric mean with geometric SD (*n* = 6–7). Statistical test: Wilcoxon paired test, not significant (ns). (**b**,**e**) The localization of PGRMC1 protein (in green) was assessed by immunocytofluorescence and nuclei were stained in blue (*n* = 10–12). (**c**,**f**) For both culture conditions (AG-205 and DMSO), PGRMC1 (24 kDa) was immunoblotted in equal amounts of cytoplasmic biomaterial and in equal amounts of nuclear biomaterial (*n* = 3). Immunoblotted histone H3 (17 kDa) and GAPDH (36 kDa) were used as loading controls for the nuclear and cytoplasmic fractions, respectively.

To better understand the mechanisms of action of AG-205 on PGRMC1 in both endometrial cell lines, we next evaluated the effect of AG-205 addition on PGRMC1 expression. In both cell lines, the addition of AG-205 had no significant effect on *PGRMC1* mRNA levels (Figure 1a,d). Because AG-205 was previously shown to trigger PGRMC1 translocation towards the nucleus [9], we also assessed PGRMC1 protein expression and subcellular localization by immunofluorescence, and by western blotting following cell fractionation. In both cells lines, PGRMC1 was essentially present in the cytoplasm and barely or not detected in the nucleus, and AG-205 addition had no detectable effect (Figure 1b,c,e,f).

### 3.2. Effects of AG-205 in Endometrial Cell Culture

**Figure 2:**
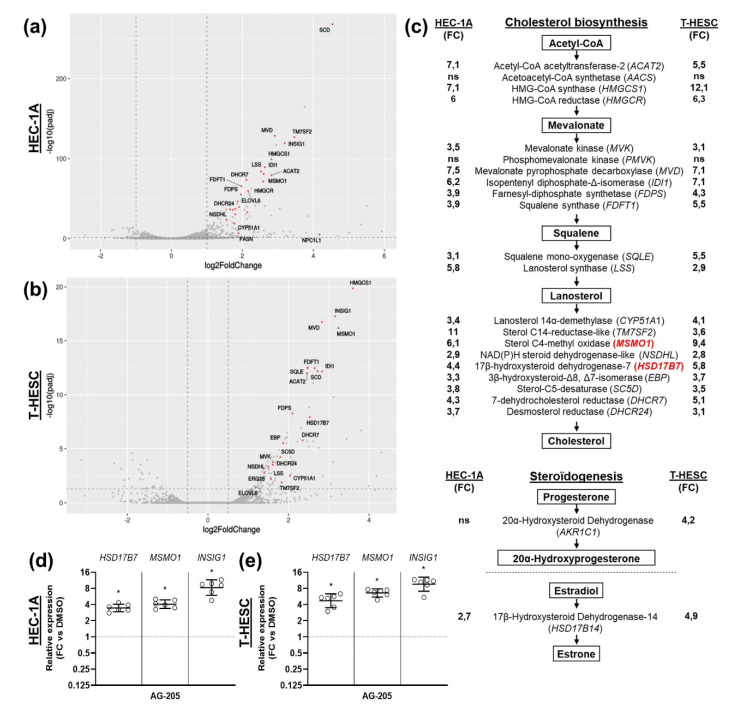
AG-205 increases RNA concentration of enzymes involved in sterol biosynthesis. RNA sequencing was used to compare transcriptomes of HEC-1A (**a**,**c**) or T-HESC cells (**b**,**c**) incubated for 32 h with 15µM AG-205 or control DMSO. (**a**,**b**) Volcano plots for HEC-1A (**a**) and T-HESC (**b**) cells were generated with Over Representation Analysis (ORA). Genes from the first GO term (GO:0016126) differentially expressed upon AG-205 addition (adjusted *p* value < 0.05 and a |log2 fold change| > 1) are represented by red dots. Only Top30 genes are labelled. (**c**) Synthetic representation of enzymes involved in cholesterol biosynthesis and steroidogenesis. Significant expression increases measured upon AG-205 addition by comparison with corresponding DMSO control are indicated as fold changes (FC) at left for HEC-1A and at right for T-HESC cells. (**d**,**e**) Relative expression of *HSD17B7*, *MSMO1* and *INSIG1* was measured by RT-qPCR in other cell samples, normalized, compared to corresponding control DMSO values, and is presented as individual fold changes (FC) in log2 scale and as geometric means with geometric SD (*n* = 6). Statistical test: Wilcoxon paired test, not significant (ns), * *p* < 0.05.

We next carried out an RNA sequencing-mediated transcriptomic comparison of cells cultured in the presence of AG-205 or with control DMSO. Volcano plots (Figure 2a,b) highlighted genes that were significantly upregulated by AG-205 addition. Interestingly, the top five Gene Ontology (GO) terms over-represented in this comparison were related to cholesterol/steroid metabolism (Table S3 and Figure S3, Supplementary Materials). More precisely, most genes coding for enzymes involved in cholesterol biosynthesis were upregulated in both cell lines (Figure 2c). The 50 genes that are most differentially expressed in both cell lines are listed in Table S4 (Supplementary Materials). This observation was attractive because previous studies have suggested a link between PGRMC1 and sterol metabolism [26,27,28,29,30]. Due to the large number of enzymes upregulated upon AG-205 addition, we hypothesized that this effect was more likely to result from modulation of one common regulator (or regulating pathway) rather than modulation of all individual genes. In this regard, insulin-induced gene 1 protein (INSIG1) stood out for several reasons. Firstly, INSIG1 is a well-known sterol regulator able to modulate several enzymatic steps in sterol metabolism (see Discussion). Secondly, INSIG1 was previously shown to directly interact with PGRMC1, although sensitivity of this interaction towards AG-205 was not addressed [30]. Thirdly, in our transcriptomic analysis, expression of *INSIG1* was strongly upregulated in both cell lines in response to AG-205. We therefore selected three genes for further experiments: *INSIG1* and two strongly upregulated enzymes of the cholesterol biosynthesis pathway, sterol C4-methyl oxidase *MSMO1* and 17β-hydroxysteroid dehydrogenase-7 *HSD17B7*, both involved in the conversion of lanosterol to cholesterol. Upregulation of the expression of these three genes upon AG-205 addition was confirmed by RT-qPCR analysis of additional cell cultures (Figure 2d,e).

### 3.3. Effects of AG-205 Are Not Mimicked by Downregulation of PGRMC1 Expression

**Figure 3:**
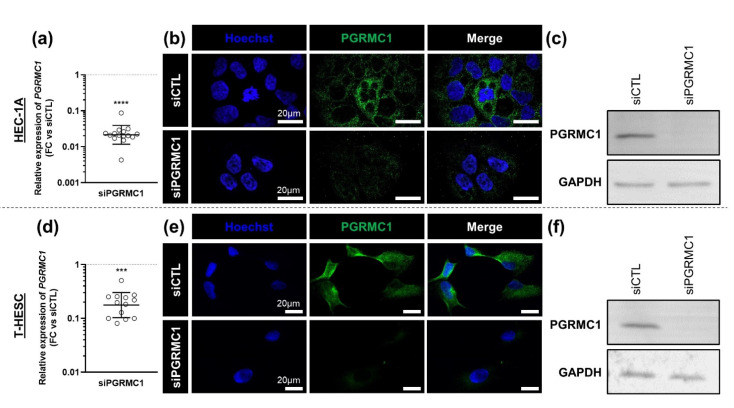
siRNA-PGRMC1 decreases expression of PGRMC1. Relative expression, immunolocalization and immunoblotting of PGRMC1 in HEC-1A (**a**–**c**) and T-HESC (**d**–**f**) cell lines treated with 10 nM of siRNA-PGRMC1 s21310 (siPGRMC1) or control siRNA-CTL (siCTL) during 80 h. (**a**,**d**) Relative expression of *PGRMC1* was measured by RT-qPCR, normalized, compared to corresponding siCTL values and is presented as individual fold changes (FC) in log scale and as geometric means with geometric SD (*n* = 13–15). Statistical test: Wilcoxon paired test, not significant (ns), *** *p <* 0.001, **** *p <* 0.0001. (**b**,**e**) Immunocytofluorescence of PGRMC1 protein (in green) and nuclear staining (Hoechst, in blue) (*n* = 4). (**c**,**f**) Immunoblot of PGRMC1 (24 kDa). GAPDH (36 kDa) was used as loading control (*n* = 3).

To directly address the contribution of PGRMC1 in the upregulation of these genes, we repeated the experiments with cells transfected with a commercial siRNA directed against *PGRMC1* mRNA (s21310). We first checked its effect on PGRMC1 expression at the mRNA and protein levels. As expected, 80 h after transfection, *PGRMC1* mRNA concentration was reduced in both cell lines (Figure 3a,d). Coherently, the PGRMC1 signal by immunofluorescence (Figure 3b,e) and immunoblotting (Figure 3c,f) were strongly reduced in both cell lines.

**Figure 4:**
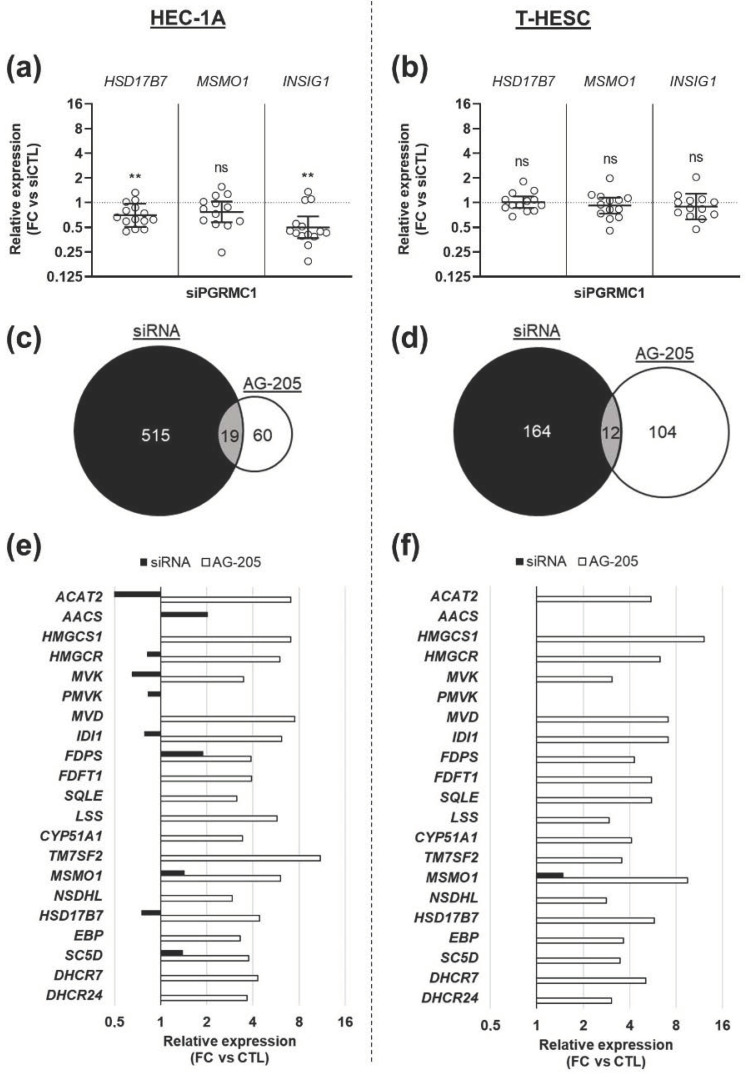
siRNA-mediated down-regulation of PGRMC1 expression does not mimic the effect of AG-205. HEC-1A (**a**,**c**,**e**) or T-HESC (**b**,**d**,**f**) cells were incubated for 80 h with 10 nM siRNA-PGRMC1 s21310 (siPGRMC1) or control siRNA-CTL (siCTL). (**a**,**b**) Relative expression of *HSD17B7*, *MSMO1* and *INSIG1* was measured by RT-qPCR (*n* = 13–14), normalized, compared to corresponding siCTL values and is presented as individual fold changes (FC) in log2 scale and as geometric means with geometric SD. Statistical test: Wilcoxon paired test, not significant (ns), ** *p* < 0.01. (**c**,**d**) Transcriptomes of siPGRMC1 and siCTL-transfected cells were established by RNA sequencing (*n* = 3) and analyzed by over-representation analysis (ORA). Results were compared with those previously obtained with AG-205 addition. Figures present the number of GO terms differentially expressed upon siPGRMC1 transfection (in black), AG-205 addition (in white) and in common (in grey). (**e**,**f**) The effect of siPGRMC1 (in black) on expression of genes represented in Figure 2c is compared to the effect of AG-205 (in white), with values retrieved from Figure 2c for direct visual comparison. Expression variations measured by comparison with corresponding control (siCTL or DMSO) are indicated as fold changes (FC).

*PGRMC1* downregulation did not reproduce the effect of AG-205 addition. Indeed, expression of the three selected genes was not significantly modified, or even slightly reduced, in cells transfected with the siRNA-PGRMC1 (Figure 4a,b).

To enlarge the scope of the comparison between the two experimental strategies (AG-205 or siRNA), we performed a new transcriptomic analysis to compare cells transfected with the siRNA-PGRMC1 or with a control siRNA. The top five most significantly enriched GO terms were totally different, in both cell lines, from those obtained with AG-205 (Table S5, Supplementary Materials). When comparing all transcriptomes together, only few GO terms were common to both strategies for each cell line (19 GOs for HEC-1A cells, Figure 4c and Table S6 (Supplementary Materials); and 12 GOs for T-HESC cells, Figure 4d and Table S7, Supplementary Materials). In contrast, several GOs were only identified in one analysis (515 GOs with the siRNA and 60 GOs with AG-205 in HEC-1A cells, Figure 4c; 164 GOs with the siRNA and 104 genes with AG-205 in T-HESC cells, Figure 4d). Most strikingly, the upregulation of genes involved in cholesterol biosynthesis was not mimicked in cells transfected with the siRNA (Figure 4e,f).

**Figure 5:**
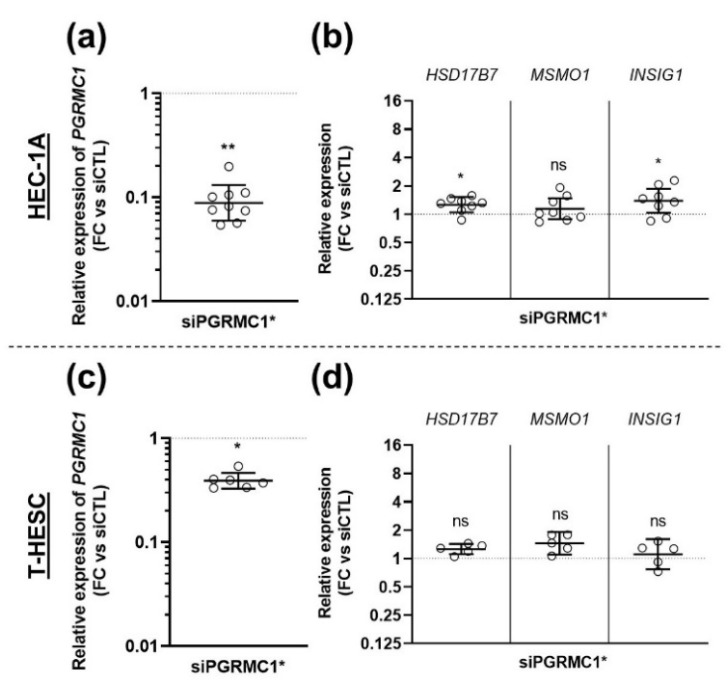
Down-regulation of *PGRMC1* expression by another siRNA does not mimic the effect of AG-205 on target genes. HEC-1A (**a**,**b**) and T-HESC (**c**,**d**) cells were incubated with 10 nM siRNA-PGRMC1 18,248 (siPGRMC1*) or control siRNA-CTL (siCTL) during 72 h. Relative expression of *PGRMC1* (**a**,**c**), *HSD17B7, MSMO1* and *INSIG1* (**b**,**d**) was measured by RT-qPCR, normalized, compared to siCTL values and is presented as individual fold changes (FC) in log or log2 scale and as geometric means with geometric SD (*n* = 5–8). Statistical test: Wilcoxon paired test, not significant (ns), * *p* < 0.05, ** *p* < 0.01.

Although accurate *PGRMC1* downregulation by the s21310 siRNA was confirmed by our validation experiments (Figure 3), we decided to transfect cells with another PGRMC1-specific siRNA. The sequence of this second siRNA-PGRMC1 (18248) was directed towards exon 1 of *PGRMC1*, a sequence common to the two isoforms of *PGRMC1* predicted in silico, whereas the siRNA-PGRMC1 s21310 was targeting exon 2 (present in only one isoform). *PGRMC1* mRNA concentration was reduced in both cell lines by siRNA 18248, although more moderately than with the first siRNA (Figure 5a,c). In agreement with data obtained with siRNA s21310, expression of the three selected genes was not modified in cells transfected with siRNA 18248, except for a marginal increase (<1.5 fold) of *HSD17B7* and *INSIG1* expression in HEC-1A cells but not in T-HESC cells (Figure 5b,d).

### 3.4. Effects of AG-205 Are Independent of PGRMC1

**Figure 6:**
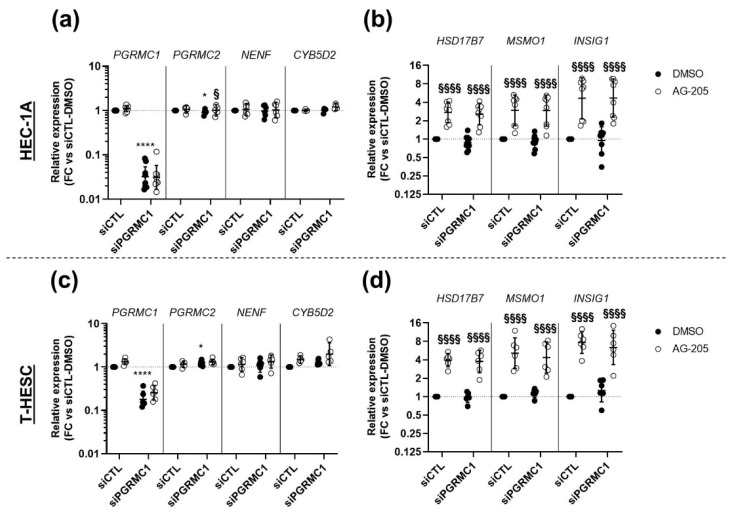
Effect of AG-205 on expression of selected target genes is not modified upon combined siRNA-mediated down-regulation of *PGRMC1*. HEC-1A (**a**,**b**) or T-HESC cells (**c**,**d**) were transfected with 10nM siRNA-PGRMC1 s21310 (siPGRMC1) or control siRNA-CTL (siCTL) during 48 h and then further incubated for 32 h with 15 µM AG-205 or DMSO. Relative expression of *PGRMC1, PGRMC2, NENF* and *CYB5D2* (**a**,**c**) and *HSD17B7, MSMO1* and *INSIG1* (**b**,**d**) was measured by RT-qPCR (*n* = 6–8), normalized, compared to dual control values (siCTL with DMSO) and is presented as individual fold changes (FC) in log or log2 scale and as geometric means with geometric SD. Statistical test: Anova-two ways with post-hoc Tukey. Statistically significant differences resulting from siPGRMC1 transfection or AG205 addition are indicated by * or §, respectively: */§ *p* < 0.05, ****/§§§§ *p* < 0.0001.

In order to further challenge the PGRMC1-dependency of AG-205 effects, we questioned whether the effects of AG-205 could be maintained after *PGRMC1* down-tuning. To this aim, the two endometrial cell lines were transfected with siRNA-PGRMC1 (s21310) or siRNA-CTL and incubated with AG-205 or its DMSO control (Figure 6). As expected, the expression of *PGRMC1* was significantly reduced in cells transfected with siRNA-PGRMC1 (Figure 6a,c). Since the MAPR family contains three other members, PGRMC2, neudesin (*NENF*) and neuferricin (*CYB5D2*), we also measured the effects of siRNA-PGRMC1 on their expression. Although *NENF* and *CYB5D2* expression was unaffected, *PGRMC2* expression was slightly, but significantly, upregulated in cells from both cell lines transfected with siRNA-PGRMC1.

In agreement with our previous experiments, expression of the three selected genes was increased upon AG-205 addition in HEC-1A cells (mean increase ~3 to 4 fold) and in T-HESC cells (mean increase ~4 to 6 fold). Most importantly, this effect was perfectly identical whether cells were transfected with the siRNA-control or the siRNA-PGRMC1, indicating that the presence of PGRMC1 was not required to mediate AG-205 response (Figure 6b,d).

### 3.5. Effects of AG-205 Are Independent of All Four MAPRs

**Figure 7:**
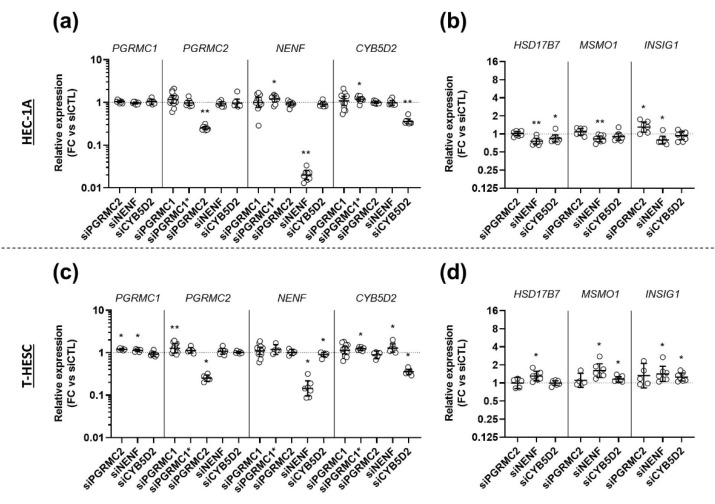
siRNA-mediated down-regulation of *PGRMC2, NENF* or *CYB5D2* expression does not mimic the effect of AG-205 on target genes. HEC-1A (**a**,**b**) and T-HESC (**c**,**d**) cells were incubated with 10 nM siRNA-PGRMC1 s21310 (siPGRMC1), siRNA-PGRMC1 18,248 (siPGRMC1*), siRNA-PGRMC2 (siPGRMC2), siRNA-NENF (siNENF), siRNA-CYB5D2 (siCYB5D2) or control siRNA-CTL (siCTL) during 72 h (*n* = 4–8). Relative expression of *PGRMC1, PGRMC2, NENF* and *CYB5D2* (**a**,**c**) and *HSD17B7, MSMO1* and *INSIG1* (**b**,**d**) was measured by RT-qPCR, normalized, compared to siRNA-CTL values and is presented as individual fold changes (FC) in log or log2 scale and as geometric means with geometric SD. Statistical test: Wilcoxon paired test, * *p* < 0.05, ** *p* < 0.01. Only differences statistically significant by comparison with the control condition (siCTL) are indicated.

**Figure 8:**
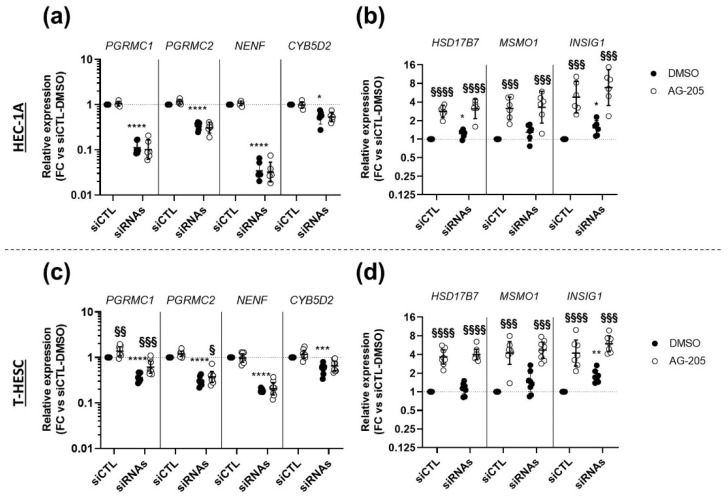
Effect of AG-205 on expression of selected target genes is not modified upon combined siRNA-mediated down-regulation of *PGRMC1, PGRMC2, NENF* and *CYB5D2* expression. HEC-1A (**a**,**b**) or T-HESC cells (**c**,**d**) were transfected with a combination of 2 nM siRNA-PGRMC1 s21310, 2 nM siRNA-PGRMC1 18248, 2 nM siRNA-PGRMC2, 2 nM siRNA-NENF and 2 nM siRNA-CYB5D2 (siRNAs) or 6 pmol control siRNA-CTL (siCTL) during 48 h. They were then further incubated for 32 h with 15 µM AG-205 or DMSO. Relative expression of *PGRMC1*, *PGRMC2*, *NENF* and *CYB5D2* (**a**,**c**) and *HSD17B7, MSMO1* and *INSIG1* (**b**,**d**) was measured by RT-qPCR (*n* = 6–8), normalized, compared to dual control values (siCTL with DMSO) and is presented as individual fold changes (FC) in log or log2 scale and as geometric means with geometric SD. Statistical test: Anova-two ways with post-hoc Tukey. Statistically significant differences resulting from siPGRMC1 transfection or AG205 addition are indicated by * or §, respectively: */§ *p* < 0.05, **/§§ *p* < 0.01, ***/§§§ *p* < 0.001, ****/§§§§ *p* < 0.0001.

We next hypothesized that AG-205 was potentially able to interact with another MAPR rather than with PGRMC1. We therefore investigated whether the effects of AG-205 could be reproduced by transfecting cells with a siRNA against one of the three other MAPR genes (Figure 7), and whether the effects of AG-205 would be maintained or not upon simultaneous down-tuning of all four MAPRs (Figure 8).

In the first set of experiments, we transfected HEC-1A cells (Figure 7a,b) and T-HESC cells (Figure 7c,d) with one siRNA directed against *PGRMC1*, *PGRMC2*, *NENF* or *CYB5D2*. At the end of the culture, we first ensured that concentration of the targeted MAPR mRNA was significantly reduced. We also measured expression of the other MAPRs to identify potential compensation on their expression (Figure 7a,c). In both cell lines, each siRNA elicited downregulation of its target by at least 3-fold by comparison with the control siRNA. Globally, the siRNAs had no, or marginal, effects on the expression of the other MAPR genes. Only a very limited (<1.3-fold mean) but significant upregulation was measured for some combinations, as indicated in the figure. Then, we tested the effect of these siRNAs on expression of the three selected genes (Figure 7b,d) and measured some significant changes, but they were marginal by comparison with the effects of AG-205. The siRNA against *PGRMC2* induced a ~1.28-fold upregulation of *INSIG1* in HEC-1A cells. Surprisingly, the siRNA against *NENF* induced downregulation of the three genes in HEC-1A cells (~1.31-fold for *HSD17B7*; ~1.19-fold for *MSMO1* and ~1.32-fold for *INSIG1*) and upregulation in T-HESC cells (~1.32-fold for *HSD17B7*; ~1.48-fold for *MSMO1* and ~1.28-fold for *INSIG1*). Similarly, the siRNA against *CYB5D2* had opposite effects on some genes in both cell lines: it induced a ~1.23-fold downregulation of *HSD17B7* in HEC-1A cells and upregulation of *MSMO1* (~1.15-fold) and *INSIG1* (~1.27-fold) in T-HESC cells. In summary, transfection with any of the three other MAPR-targeting siRNAs did not reproduce the magnitude of the effects of AG-205.

In the second series of experiments, in an approach similar to that used in Figure 6, we transfected cells with the siRNA control or with a mixture of the 5 siRNAs, simultaneously targeting all four MAPR mRNAs (including the 2 siRNAs against *PGRMC1*) before adding AG-205 or control DMSO. The efficiency of the simultaneous downregulation of *PGRMC1*, *PGRMC2*, *NENF* and *CYB5D2* was verified by measuring the expression of these genes in HEC-1A (Figure 8a) and T-HESC (Figure 8c). In the two cell lines, a significant decrease in expression of all four genes was observed in cells transfected with the mixture of siRNAs by comparison with cells transfected with the siRNA-CTL, both in the presence of AG-205 or control DMSO.

The addition of AG-205 had no effect on the expression of the MAPR genes (comparison between black and white symbols in Figure 8a,c), except for two minor changes in T-HESC cells: For *PGRMC1* with both siRNA-CTL and anti-MAPR siRNA mixture, and for *PGRMC2* with anti-MAPR siRNA mixture only. The siRNA mixture also induced a slight increase in *HSD17B7* in HEC-1A cells and in *INSIG1* in both cell lines (Figure 8b,d).

As expected, AG-205 significantly increased the expression of the three selected genes *HSD17B7*, *MSMO1* and *INSIG1* in the two endometrial cell lines (Figure 8b,d) and, most importantly, this effect was maintained with a similar extent in cells transfected with the siRNA mixture against all four MAPR genes. Altogether, our results indicate that neither PGRMC1, nor any of the three other MAPRs is involved in AG-205-mediated increase in the expression of genes involved in the cholesterol biosynthesis and steroidogenesis pathways in endometrial cells.

## 4. Discussion

In the present study, we compared the effects of AG-205 addition and *PGRMC1* downregulation in the culture of endometrial cell lines.

Before turning to transcriptomic analysis, we optimized AG-205 concentration and incubation time, and addressed its potential effects on cell viability and PGRMC1 expression and subcellular localization. AG-205 was rarely added alone in cell culture medium in other studies because it was essentially used to address PGRMC1 contribution to the effect of another inducer. However, it was previously shown that cell viability is reduced in various cell types with AG-205 concentrations above 20 µM: reduction by about 40% and 60% in MDA-MB-231 breast cancer cells at 20 µM and 40 µM AG-205, respectively (Ahmed, 2010); reduction by about 25%, 42% and 50% after 24 h in lung cancer-derived stem cells at 25 µM, 50 µM and 100 µM AG-205, respectively [31]. This is fully compatible with our measures of cell viability in both endometrial cells lines and supports our choice to further use 15 µM AG-205. Throughout our experiments, AG-205 had, in general, no effect on the expression of *PGRMC1* or any other MAPR, although a marginal increase in *PGRMC1* expression was occasionally measured. Moreover, 15 µM AG-205 did not allow detection of increased PGRMC1 nuclear localization, unlike previously reported in human ovarian cells with 50 µM AG-205 [9].

In both tested cells lines—T-HESC cells from fibroblastic origin and HEC-1A from epithelial origin—the most striking effect of AG-205 highlighted by our transcriptomic analyses was increased mRNA concentration of several enzymes involved in cholesterol biosynthesis, the sterol-sensitive regulator *INSIG1* and specific enzymes involved in steroidogenesis. Our results are in global agreement with the reported effects of AG-205 in the culture of primary stromal cells induced to decidualize in response to combined estradiol and progesterone [14]. However, these effects were produced in the absence of progesterone, suggesting that they are not relevant to decidualization, and, most importantly, they were not mimicked by siRNA-mediated down-regulation of *PGRMC1* or any other related MAPR (*PGRMC2*, *NENF* or *CYB5D2*). Most strikingly, the upregulation of three illustrative genes in response to AG-205 addition was fully preserved when cells were concomitantly transfected by siRNA against *PGRMC1* or all four MAPRs. We thus show for the first time that changes in expression of this set of genes in endometrial cells in response to AG-205 addition are not mediated and do not depend on PGRMC1 or any other MAPR.

However, our study does not rule out that AG-205 could (in)directly interfere with molecular mechanisms involving PGRMC1 to explain previous publications. For instance, AG-205 was recently shown to influence PGRMC1 interactions with the actin cytoskeleton in MIA PaCa-2 cells [32]. Moreover, in some studies, the downregulation of *PGRMC1* expression generated effects similar to those induced by AG-205 addition. For instance, EGFR is known to form complexes with PGRMC1, in a PGRMC1 dimer-dependent manner [8,33] and both experimental strategies (siRNA and AG-205) led to reduced EGFR levels in breast cancer cells [8]. Unfortunately, additional and more specific approaches were not used in all publications reporting effects of AG-205, and their conclusions about PGRMC1 involvement should therefore be considered with great caution. This is, for instance, illustrated with another study focusing on a link between PGRMC1, EGFR and estradiol [11]. The addition of AG-205 blocked the inhibitory effect of estradiol on zebrafish oocyte maturation, but the contribution of PGRMC1 to this mechanism and whether AG-205 did not modify estradiol concentration by modulating expression of metabolic enzymes remain to be confirmed. This would be extremely instructive since a link between PGRMC1, estradiol/estradiol receptor and breast cancer progression was evidenced in other reports that relied on downregulation [34] or overexpression [29] of PGRMC1, and are therefore not challenged by our study.

These and other examples clearly highlight that, although additional work is required to address its actual mechanisms of action, AG-205 should no longer be referred to, and commercialized as a specific inhibitor/ligand of PGRMC1, and studies based on the use of AG-205 should be replicated with an alternative approach specifically targeting PGRMC1, even when PGRMC1 downregulation is not considered an appropriate choice. For instance, progesterone is known to inhibit GnRH neurons and this effect was abolished by the concomitant addition of AG-205, but not by mifepristone, a progesterone antagonist acting on the canonical nuclear progesterone receptor [35]. The authors concluded that progesterone was therefore acting through PGRMC1 as an alternative progesterone receptor. Unfortunately, experiments with siRNA were disregarded because of heterogeneity in the GnRH neuron population. Potential changes in progesterone concentration were not monitored after AG-205 addition, and the precise contribution of PGRMC1 remains unclear.

Despite its lack of specificity towards PGRMC1, AG-205 may remain attractive for its anti-mitotic, anti-migratory and anti-invasive activities [10] which stimulated hopes of therapeutic applications, as illustrated by the patent targeting breast cancer [13]. However, our data clearly highlight potential detrimental endometrial side-effects, by indicating that its use could modulate endometrial concentration of estradiol and progesterone through local metabolism/intracrinology [36]. Endometrial pathologies result from a hormonal imbalance between estradiol and progesterone. For instance, endometriosis is characterized by localisation of endometrial tissue at ectopic sites outside the uterus. Progesterone and synthetic progestins are efficiently used to inhibit estrogen-dependent progression of the lesions. Unfortunately, in one-third of the patients, progestogens fail to limit the expansion of the lesion [37,38]. A decrease in expression of the nuclear progesterone receptors in stromal ectopic cells was established [39], but not systematically observed [40] and, therefore, is not sufficient to explain progesterone resistance. The contribution of mPRs and MAPRs is currently investigated. However, our results suggest that therapeutic administration of AG-205 to women (for instance against breast cancer) could lead to increased estradiol concentration and/or decreased progesterone concentration, thereby favoring endometriosis development and progression, and increasing the risk of endometrial hyperplasia and cancer development.

In conclusion, although AG-205 may seem attractive for the development of new therapeutic strategies due to its various effects, in particular against cancer progression, it should no longer be considered as a PGRMC1 inhibitor and its precise mechanisms of action and potential detrimental side-effects in medical use should be carefully investigated and documented in the future.

## Data Availability

RNA sequencing data of cells cultured with AG-205 are stored in NCBI under GEO accession number GSE174305. RNA sequencing of cells cultured with the siRNA-PGRMC1 (s21310) was only used for the purpose of comparison and will be commented in detail in another publication. The transcriptomes of siRNA-transfected cells are only available from the corresponding author on reasonable request. The Supplementary Materials are available online in FigShare at https://figshare.com/s/256ad96e895af6eaab9c, accessed on 26 May 2021 [41].

## Supplementary Materials

The following are available online at https://figshare.com/s/256ad96e895af6eaab9c [41], Table S1: siRNAs used for siRNA-mediated down-regulation, Table S2: Sequences of primers for specific amplification by quantitative real-time PCR, Table S3: Top5 GO terms identified by over-representation analysis (ORA) in HEC-1A and T-HESC cells cultured in the presence or absence (DMSO) of AG-205. Terms are sorted by adjusted *p* value (padj), Table S4: Top50 genes differentially expressed upon AG-205 addition in HEC-1A and T-HESC cells. Genes are sorted by adjusted *p* value (padj), Table S5: Top5 GO terms identified by over-representation analysis (ORA) in HEC-1A and T-HESC cells transfected with siPGRMC1 or siCTL. Terms are sorted by adjusted *p* value (padj), Table S6: Common GO terms identified by over-representation analysis (ORA) in transcriptomes of HEC-1A cells upon siPGRMC1 transfection or AG-205 addition, Table S7: Common GO terms identified by over-representation analysis (ORA) in transcriptomes of T-HESC cells upon siPGRMC1 transfection or AG-205 addition, Figure S1: Chemical structure of AG-205, Figure S2: Impact of AG-205 on cell viability, Figure S3: Ancestor chart of top5 GO terms identified by over-representation analysis (ORA) in HEC-1A and T-HESC cells cultured in the presence or absence (DMSO) of AG-205.
